# Diagnosis of infantile myofibromatosis with pseudo-ulcerated plaque using prenatal ultrasound: A case report

**DOI:** 10.3892/etm.2014.2010

**Published:** 2014-10-08

**Authors:** FEIXUE ZHANG, DONGFENG CHENG, MEI WU, LING GE, XIANGXING MA

**Affiliations:** 1 Department of Radiology, Division of Ultrasound, The Second Hospital of Shandong University, Jinan, Shandong 250012, P.R. China; 2 The Fourth People’s Hospital of Jinan City, Jinan, Shandong 250012, P.R. China; 3 Department of Radiology, Qilu Hospital of Shandong University, Jinan, Shandong 250012, P.R. China

**Keywords:** infantile myofibromatosis, pseudo-ulcerated plaque, ultrasound, magnetic resonance imaging, prenatal

## Abstract

The present case report describes a case of infantile myofibromatosis (IM) with a pseudo-ulcerated plaque on the right side of the back of a fetus, detected in the 38th week of gestation using prenatal ultrasound. The fetus was examined weekly by ultrasound to measure the size of the mass. At birth, the scarlet mass was slightly elevated compared with the skin around it, with a cavity in the center. It appeared similar to an ulcerated plaque, but the surface of the mass was intact and smooth with a stratum lucidum. Thus, the mass was indicated to be a pseudo-ulcerated plaque. Three months later, the mass had grown larger and so was removed by surgery. The pathology of the mass was confirmed as IM. It is suggested that IM should be considered when a soft tissue tumor is presented by prenatal ultrasound.

## Introduction

Infantile myofibromatosis (IM), a rare benign neoplasm with an incidence of 1 in 400,000 (1), can occur at any organ, particularly the skin or subcutaneous tissues or muscles (2,3). IM is the most common fibrocellular tumor in infancy and childhood (4–6). The features of the disease are painless, solitary and congenital lesions (3). The diagnoses of IM by prenatal ultrasound have been reported in seven previous studies (7–13); however, its diagnosis by prenatal magnetic resonance imaging (MRI) has been described in only one previous report (11). In addition, IM with ulcerated plaque has been described in three case reports (2,14,15). The present case report describes a case of IM with a pseudo-ulcerated plaque that, to the best of our knowledge, has never been reported before.

## Case report

**Figure 1:**
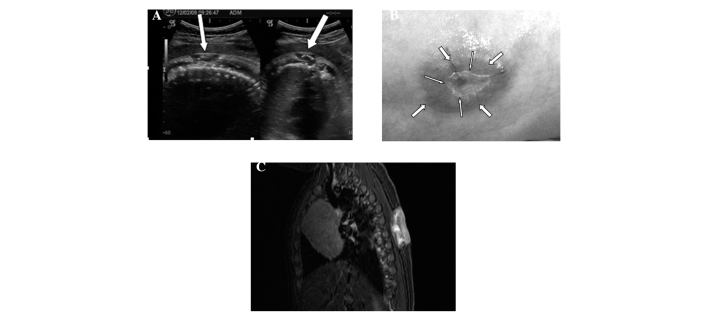
(A) Prenatal ultrasonogram showing a mass on the right side of the back of a fetus in the 38th week of gestation. The lesion was hypoechoic with calcification and a clear boundary (white arrows). (B) Image of the mass at birth. The small arrows indicate the cavity in the center of the mass, and the large arrows indicate the pseudo-ulcerated plaque. (C) Magnetic resonance image obtained three months after birth showing heterogeneous high short time inversion recovery signal with clear boundary, which implicates the right paraspinal muscle, but no connection with the canalis spinalis.

A 30-year-old female, gravida 1 para 1, was examined conventionally by prenatal ultrasound in the 38th week of gestation. The study was approved by the Medical Ethics Board of The Second Hospital of Shandong University (Jinan, China). The patient’s parents signed a statement of informed consent. A mass (2.4×2.2×1.1 cm in size) was discovered on the fetal right back (T3-8), which was hypoechoic with sporadic hyperecho (Fig. 1A). Color Doppler flow imaging (CDFI) showed intermittent blood flow inside and around the mass. On the next day, MRI examination showed a normal result. The woman gave birth to a boy in the 41st week of gestation. A scarlet mass that was slightly elevated compared with the skin around it with a cavity in the center, was observed on the right side of the back of the newborn at birth. These characteristics suggested that the mass was an ulcerated plaque; however, the mass was not actually an ulcerated plaque according to the visual inspection. The surface of the mass was white and intact, and was indicated to be a pseudo-ulcerated plaque (Fig. 1B). Physical examinations showed no abnormality. Considering that the mass was congenital and might regress spontaneously, doctor suggested that the parents should wait.

**Figure 2:**
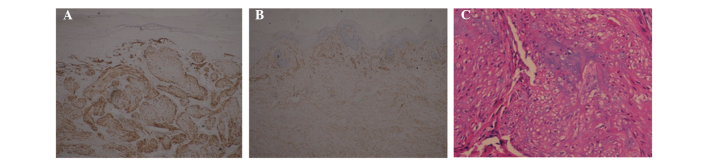
Immunohistochemical analyses showing (A) immunopositivity for smooth muscle actin (SMA; magnification, ×40), (B) immunopositivity for vimentin (VIM; magnification, ×40), and (C) myofibroblasts following hematoxylin and eosin staining (magnification, ×200).

Three months later, the mass had increased in size, and reached a size of 5.0×4.0×3.5 cm. It showed a clear boundary, but no tenderness or activities. Ultrasound investigation showed a subcutaneous hypoechoic tumor (3.8×3.2×2.4 cm) on the right side of the back with a clear boundary and heterogeneous echogenicity inside. In addition, calcification, an echoless region and invasion of the erector spinae muscles were observed. CDFI showed intermittent blood flow inside and around the tumor. MRI demonstrated the presence of a mass under the subcutaneous soft tissue on the right side of the back between T3-8 with a heterogeneous or long T1 signal, equal or long T2 signal, and heterogeneous high short time inversion recovery (STIR) signal, implicating the right paraspinal muscle, but not canalis spinalis (Fig. 1C). Three days later, the mass was resected by surgery and sent for pathological study. The surgery revealed that a tumor with a size of 5.0×4.0×3.0 cm was present under the subcutaneous soft tissue, with a clear boundary and invasion of erector spinae muscles. The mass was confirmed as IM, with rare nuclear fission and unclear cell atypia. The results of immunohistochemistry were as follows: smooth muscle actin (+) and vimentin (+). Hematoxylin and eosin staining showed that the myofibroblasts were arranged into a clustered or circinate structure (Fig. 2). Two years later, the patient was healthy with no recurrence of IM.

## Discussion

IM is a type of rare mesenchymal tumor that originates from myofibroblasts, with 88% IM patients being younger than 2 years old (16). Male patients account for 60.8% of all IM cases, while female patients account for 39.2% (3). IM is solitary or multicentric according to the number of lesions (3). The solitary form of IM usually occurs in the skin, subcutaneous tissues, muscles and the skull, with good prognosis (3,16–19). By contrast, the multicentric form of IM widely invades subcutaneous muscles, bones and viscera (1,19,20). Both solitary and multicentric forms of IM are associated with poor prognosis if they affect viscera, particularly the heart, the lungs and gastrointestinal tracts (9,13,18). Ultrasound imaging characteristics of IM include stiffness, encapsulation, calcification, liquefaction and signs of reduced blood flow (4,21). To the best of our knowledge, a case of IM with a pseudo-ulcerated plaque has never been reported in any previous literature. The case of IM with pseudo-ulcerated plaque sign discussed in the present report was solitary, and involved skin, subcutaneous tissues and muscles. However, the patient exhibited a good prognosis. The pathology of the white and transparent surface was diagnosed as a cuticular layer. The imaging features of the tumor shown by ultrasound, including a clear boundary, calcification, liquefaction and two or three strips of blood flow signals, were consistent with previous literature (4). The infant was examined by MRI prior to birth and surgery. MRI examination before birth did not show the mass, probably because the tumor was too small to be detected or the posture of the fetus was not appropriate for examination. Although IM is congenital and the lesions usually regress spontaneously in one or two years (18), the IM in the present case grew larger after three months and thus surgery was performed. The surgery may have been avoided if the newborn had been given a long-term follow-up (3).

While IM can be diagnosed by prenatal ultrasound (7–13), it is important to differentiate IM from hemangioma, neurofibroma or desmoids (5). Although it is difficult to definitely diagnose IM using prenatal ultrasound, it is possible to confirm the location and numbers of IM lesions. If the tumor is single and located in superficial organs, pregnancy can continue; if it is not single and has infiltrated into other important viscera, the pregnancy should be terminated. In summary, prenatal ultrasound can be used to detect fetal lesions and to diagnose IM according to its imaging features.
